# Reviews and Bibliographical Notices

**Published:** 1867-03

**Authors:** 


### REVIEWS AND BIBLIOGRAPHICAL NOTICES.

---

*An Index of Diseases and their Treatment.* By THOMAS HAWKES TANNER, M. D., F. L. S., member of the Royal College of Physicians at Philadelphia. Lindsay & Blackinstone, 1865.

This is an excellent work, and one that the active practitioner will find a most valuable addition to his library. The author's description of the character, symptoms, and treatment of the diseases discussed, is concise and comprehensive. The great object of the volume is to facilitate the daily work of the practitioner. Every active practitioner should have this little volume.

*A Handy-Book of Ophthalmic Surgery, for the use of Practitioners.* By JOHN Z. LAWRENCE, F. R. C. S., M. B. (Univ. London) Surgeon to the Ophthalmic Hospital, etc.. etc., and ROBERT C. MOORE, House Surgeon to the Ophthalmic Hospital, Southwark; with numerous illustrations. Philadelphia: Henry C. Lea. 1867.

To those who desire to keep pace with the rapid and wonderful improvements in Ophthalmic Surgery, and have no time to read the ponderous contributions to this department of our science, would do well to procure this work. The author says: "In writing these pages, it has been our aim to bring the principles and practice of Ophthalmic Surgery within a small compass, to supply the wants of the busy practitioner, who may have neither time nor opportunity to read the innumerable contributions that Ophthalmic Surgery and Science have received within the last fifteen years."

We have read this little volume with great pleasure, and could not be induced, for thrice the published price, to be without it.

There are several other publications received, but as we have not had the leisure to look through them, we will defer any notice until our next issue.

